# Reported *Drosophila* courtship song rhythms are artifacts of data analysis

**DOI:** 10.1186/1741-7007-12-38

**Published:** 2014-06-26

**Authors:** David L Stern

**Affiliations:** 1 Janelia Farm Research Campus, Ashburn VA 20147, USA

**Keywords:** Artifacts, Biological rhythms, Courtship song, *Drosophila*

## Abstract

**Background:**

In a series of landmark papers, Kyriacou, Hall, and colleagues reported that the average inter-pulse interval of *Drosophila melanogaster* male courtship song varies rhythmically (KH cycles), that the *period* gene controls this rhythm, and that evolution of the *period* gene determines species differences in the rhythm’s frequency. Several groups failed to recover KH cycles, but this may have resulted from differences in recording chamber size.

**Results:**

Here, using recording chambers of the same dimensions as used by Kyriacou and Hall, I found no compelling evidence for KH cycles at any frequency. By replicating the data analysis procedures employed by Kyriacou and Hall, I found that two factors - data binned into 10-second intervals and short recordings - imposed non-significant periodicity in the frequency range reported for KH cycles. Randomized data showed similar patterns.

**Conclusions:**

All of the results related to KH cycles are likely to be artifacts of binning data from short songs. Reported genotypic differences in KH cycles cannot be explained by this artifact and may have resulted from the use of small sample sizes and/or from the exclusion of samples that did not exhibit song rhythms.

## Background

**Figure 1:**
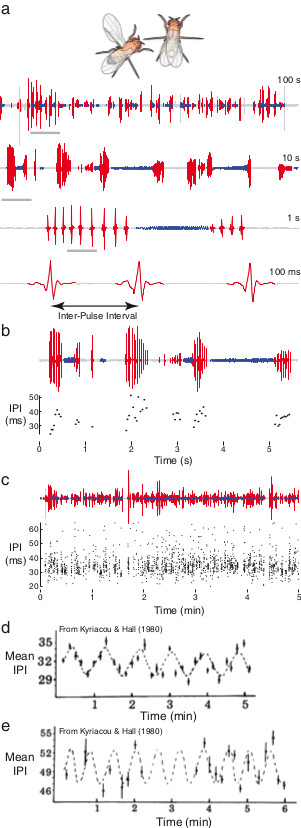
**Courtship song and putative courtship song rhythms. (a)** An example of a segmented single recording is shown at multiple scales, from 100 s to 100 ms. Each subsequent clip is derived from the region highlighted with the grey bar. A single inter-pulse interval (IPI) is illustrated in the 100 ms clip. **(b)** The IPI varies over an order of approximately two on a short timescale, even within a single pulse train. The example song trace is shown above and the IPI values are shown below. **(c)** The IPI displays similar high variation over a 5 min interval. The song trace is shown above and the IPI data below. **(d, e)** Examples of KH cycles produced by *D. melanogaster***(d)** and *D. simulans***(e)** reported in reference [6].

Males of the vinegar fly, *Drosophila melanogaster*, flap one wing at a time to sing a courtship song to females [1,2] (Figure 1a). This song consists of trains of an approximately sinusoidal ‘sine song’ and a series of discrete pulses, called ‘pulse song’, often concatenated into song bouts [3] (Figure 1a). The time interval between individual pulses, called the inter-pulse interval (Figure 1a), tends toward a characteristic interval for each species of *Drosophila* that sings pulse song, and in *D. melanogaster* this inter-pulse interval is approximately 35 to 40 ms. However, the inter-pulse interval is variable at multiple time scales (Figure 1b,c): within a single train of pulses, between trains of pulses, between different individuals of a strain, and between different strains [3-5].

In 1980, Kyriacou and Hall [6] reported that, on average, the inter-pulse interval cycled with an amplitude of approximately 3 ms and a period of approximately 1 min (0.017 Hz) in *D. melanogaster* (Figure 1d) and 30 s (0.03 Hz) in *D. simulans* (Figure 1e). Following tradition, I will call this periodicity KH cycles. In the same paper, they reported that mutations in the *period* locus that increased, decreased, and ablated periodicity of the circadian rhythm had similar effects on KH cycles. Later papers reported that transformation of the *period* locus into individuals carrying a null allele of the *period* gene restores KH cycles to arrhythmic males [7]; that females of *D. melanogaster* and *D. simulans* mate faster when presented with artificial songs that contain the species-specific inter-pulse interval and KH cycles than when presented with heterospecific songs [8]; that the difference in KH cycles between *D. melanogaster* and *D. simulans* is controlled by a locus or loci on the X chromosome (where the *period* gene resides) [9]; that hybrid females of *D. melanogaster* and *D. simulans* prefer songs with a combination of intermediate inter-pulse interval and KH cycles [9]; that *D. simulans* KH cycles can be transferred into *D. melanogaster* by genetic transformation of the *D. simulans period* locus into a *D. melanogaster* strain carrying a *period* null mutation [10]; and that this effect of the *period* locus can be mapped to a 700 bp region of the largest exon of *period*, which contains a threonine-glycine repeat region that differs in length between *D. simulans* and *D. melanogaster*[10].

This series of papers has been heavily cited and discussed in the secondary literature, in part because this is one of only two examples of a species-specific behavioral difference that has been traced to a genetic difference. (Variation in vasopressin V_1A_ receptor expression likely contributes to differences in affiliative behavior between vole species [11,12]). Given the importance of this work, it would be of great interest to follow-up on these initial studies and to identify the molecular mode of action of *period* in courtship song cycles. However, no other genes have been implicated in control of KH cycles and the molecular mechanism by which variation in the *period* locus influences KH cycles has not been identified. In fact, there have been no papers published since 1991 providing further evidence for the mode of action of the *period* gene in courtship song rhythms. In particular, there is still no satisfactory explanation for how the *period* gene, whose expression cycles approximately diurnally and whose protein product is degraded during daylight hours [13], could contribute to rhythms that cycle on the order of 30 to 60 s. This fact contrasts strongly with the significant progress over the past 20 years revealing the molecular basis for circadian rhythms [14,15].

There are two possible reasons for the recent absence of published work on this problem. First, the tools available over the past 20 years may have been insufficient to allow more detailed investigation. For example, tools to allow high throughput analysis of courtship song and detailed analysis of neural circuits in *Drosophila* have only recently become available [3,16]. Second, it is possible that KH cycles are not real phenomena, but instead are an artifact of the methods of data collection or analysis utilized in earlier papers. This possibility was raised originally in the late 1980s, when two independent groups reported that they were unable to detect KH cycles [17,18]. (Also see [19-22]). In addition, my colleagues and I were unable to detect significant evidence for KH cycles in five wild-type strains of *D. melanogaster*[3]. However, one independent group has reported that they can detect KH cycles [23]. As statisticians have noted, it is likely that in most of the early studies too few pulse events were sampled to allow robust estimates of rhythms with periods on the order of 60 s [24]. Nonetheless, there has been no reasonable explanation for the fact that Kyriacou and Hall and at least one other group have consistently found evidence for KH cycles.

In apparent further support for the existence of Kyriacou and Hall rhythms, Ritchie and colleagues showed that female *D. melanogaster* prefer synthesized courtship song containing KH cycles over song that does not contain this rhythmicity [25]. This may imply that females have the ability to detect KH cycles in courtship song. However, there is an alternative explanation for their observations. Normal *D. melanogaster* song contains complex, non-random dynamics of the inter-pulse interval on the scale of seconds [3] (Figure 1b). Ritchie *et al*. compared synthesized song that contained KH cycles with synthesized song containing either randomly patterned inter-pulse intervals, constant inter-pulse intervals, or silence. Remarkably, females showed very similar preferences for randomly patterned song, constant song, and silence, even though females are known to prefer song over silence [26]. It is possible that the randomly patterned song and constant inter-pulse interval song in this experiment did not accurately mimic wild-type song. Most importantly, this experiment is missing an additional critical control, song with natural patterns of inter-pulse intervals. Without this control, it is premature to conclude that females prefer song containing KH cycles to song without cycles. It is possible that *D. melanogaster* females prefer some modulation of inter-pulse interval over song with a constant inter-pulse interval — since variation in the inter-pulse interval is a common feature of real song — but this has not yet been demonstrated.

**Figure 2:**
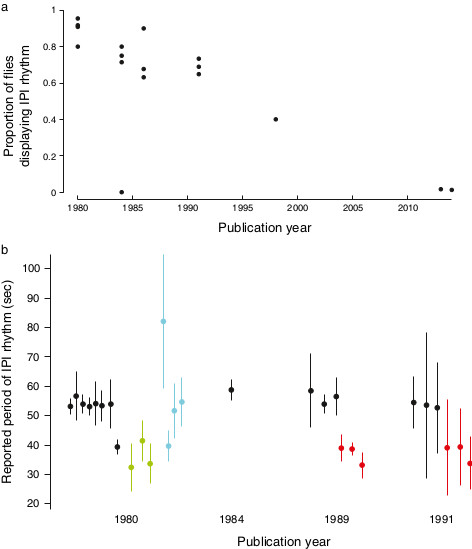
**History of reported values for KH cycles in *Drosophila *species and mutants. (a)** The proportion of animals that were reported to display ‘significant’ KH cycling has declined on average over consecutive publications. Note that different methods were employed to detect rhythms in different studies and statistical significance was assessed in different ways in different studies. The data from 2013 is from a publication that includes the current author and the data from 2014 are from this publication. **(b)** Estimates of the distribution of the period of KH cycles from publications from four years. Dots represent the mean and bars represent twice the standard deviation, back calculated from the reported standard errors of the mean and sample sizes. The bars thus include approximately 95% of the estimated song rhythms for each sample. Different colors represent songs from animals with different genotypes: Black are *D. melanogaster* wild type, red are *D. simulans* wild type, green are *period*^*short*^, blue are *period*^*long*^. IPI, inter-pulse interval.

It is worth noting that, in all studies on this subject, investigators have reported that KH cycles can be detected in some, but not all, individuals. Songs not displaying rhythms were not included in further analysis. Curiously, the proportion of positives detected in each study has declined steadily since the initial report (Figure 2a, Additional file 1: Table S1). This trend exists in the face of reportedly improved methods for detecting KH cycles [22,23]. Estimates of the periodicity of KH cycles vary somewhat between reports, but most values fall between approximately 25 and 65 s, corresponding to frequencies of 0.04 to 0.0154 Hz (Figure 2b). While the original reports appear to demonstrate strong differences between different genotypes (in part by plotting standard errors of the means), reconstruction of the 95% confidence intervals for the original data ranges illustrates that the different genotypes exhibit considerable overlap (Figure 2b, Additional file 2: Table S2).

Since KH cycles are a fascinating phenomenon with implications for speciation and evolutionary biology, I had hoped to follow-up on earlier reports to study the molecular and neurobiological mechanisms regulating KH cycles. Despite efforts to match earlier experimental conditions as closely as possible, I was unable to detect KH cycles. As I show, however, non-significant rhythms of the inter-pulse interval in the frequency range reported by Kyriacou and Hall are generated by binning the data of short songs. These results suggest that KH cycles do not exist and that previous reports reflect artifacts of data analysis.

## Results

I searched for KH cycles first by attempting to replicate the experimental paradigm used by Kyriacou and Hall as closely as possible. Unlike many earlier attempts, I employed recording chambers with dimensions that matched those used by Kyriacou and Hall. I recorded song from wild-type *D. melanogaster* and *D. simulans* flies and mutants of the *period* locus (null, *short* and *long*). I recorded each courting male for approximately 45 min and, to avoid possible artifacts arising from limited sample sizes, I discarded recordings that included fewer than 1,000 pulses.

Testing for rhythmicity in time series of biological data presents multiple challenges [27,28]. One classical method for detecting rhythmicity is to calculate the Fourier transform of the time series, which essentially transforms the time series into the frequency domain by representing the original wave form as the sum of a series of sinusoids of different frequencies, power, and phase. Power can then be plotted for each frequency in the form of a periodogram and periodicity can be detected as peaks of strong power. However, the Fourier transform requires equally spaced data. With unequally spaced data, or data sampled regularly but with missing values, the Fourier transform does not have well-defined properties and can generate biased periodograms [27,29]. Furthermore, the statistical significance of values in these periodograms cannot be determined. Everyone who has worked on KH cycles was aware of, and made efforts to ameliorate, these limitations of classical periodogram analysis [23,30].

To overcome these difficulties, astronomers developed methods based on the least-squares fit of a series of sinusoids of different frequencies to the data [29,31]. This method, now called the Lomb-Scargle periodogram, does not require regularly sampled time series and, because the power at different frequencies represents the contribution of that frequency to the variance in the signal, it allows estimation of the statistical significance of the power at a particular frequency [29,31]. The Lomb-Scargle periodogram is now widely recognized as a powerful method for detecting rhythmicity in biological time series [27,28,32-34]. In addition, we have shown previously through simulation [3] that the Lomb-Scargle periodogram is very sensitive to potential KH cycles, with power greater than 80% to detect putative KH cycles when the signal-to-noise ratio is at least one (Additional file 3: Figure S1). Therefore, I used the Lomb-Scargle periodogram to search for rhythmicity in the inter-pulse interval.

**Figure 3:**
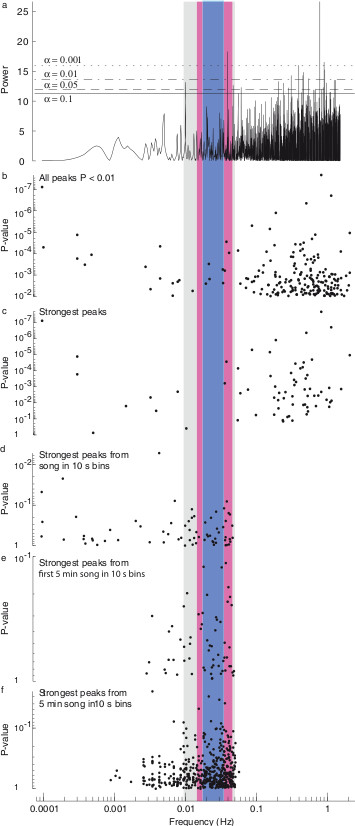
**Lomb-Scargle periodogram analysis of inter-pulse interval time series. (a)** Lomb-Scargle periodogram of a single 45-min time series from a Canton-S male. Power at frequencies ranging from approximately 0.0001 to 2 Hz is shown. Horizontal lines representing significance values at alpha equal to 0.1, 0.05, 0.01, and 0.001 are shown and labeled. This recording displayed numerous peaks significant at alpha <0.01 in the range of approximately 0.1 to 1 Hz and one peak significant at this level at approximately 0.03 Hz. The high-frequency peaks represent modulations in the inter-pulse interval on the order of seconds, which are observed often in male courtship song (compare with Figure 1b). **(b-f)** Summary of Lomb-Scargle periodogram results over all recordings of *D. melanogaster* Canton-S males. **(b)** The P-values for all peaks in the Lomb-Scargle periodograms significant at the 0.01 level and below. **(c-f)** The P-values of only the single strongest peak from the Lomb-Scargle periodogram of each recording **(c)**, for songs binned into 10 s intervals **(d)**, for the first 5 min of song binned into 10 s intervals **(e)**, and for songs divided into 5 min recordings and binned into 10 s intervals **(f)**. In all panels, frequency, in Hz, is displayed along the X axis. The vertical color bars represent the range of KH cycles for *D. melanogaster* from the 1980 publication (blue), for all *D. melanogaster* and *D. simulans* published values from 1980 to 1991 (red), and for all genotypes from 1980 to 1991 (grey), respectively (compare with Figure 2b).

**Figure 4:**
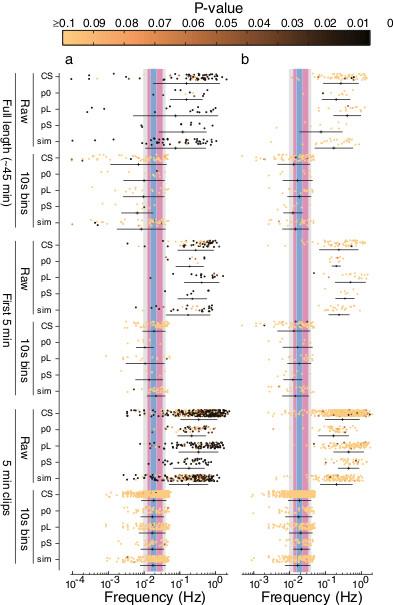
**Lomb-Scargle periodogram analysis of multiple genotypes.** Each datum represents the frequency with maximum power in a single song. The P-value for each value is pseudocolored according to the colorbar shown along the top of the figure. The mean ± 1 standard deviation is shown below each cloud of points. Each row contains data from a different genotype (*D. melanogaster* Canton-S (CS), *period* [*Null*] (p0), *period* [*Long*] (pL), *period* [*Short*] (pS), or *D. simulans* (sim)) analyzed in different ways. On the top, full-length (45 min) recordings were analyzed either as raw inter-pulse interval data or with data summarized as the means of 10 s bins. In the middle, the first 5 min after a male started singing were analyzed for each recording. This mimics most closely the analyses performed in most previous studies. On the bottom, full-length songs were divided into 5 min clips and each clip was analyzed separately. **(a)** The full-length raw data for all genotypes displayed significant rhythms across a wide frequency range on the order of seconds. These high-frequency signals are retained in the 5 min clips. Binning the data truncates the high frequency range that can be sampled, eliminating the statistically significant high frequency rhythms. The remaining maximum Lomb-Scargle periodogram peaks cluster near the previously reported range for KH cycles (illustrated with blue, red, and grey bands, as in Figure 3), although most of these values are not statistically significant. **(b)** Randomized song data display similar patterns as the real data, except that the high frequency rhythms in randomized data are mostly not statistically significant.

An example of a Lomb-Scargle periodogram for a single Canton-S male is shown in Figure 3a. The statistically significant peaks (*P* <0.01) in the Lomb-Scargle periodograms across all Canton-S flies are shown in Figure 3b. It is clear that many time series contain evidence for relatively high frequency rhythms (on the order of seconds), but relatively little evidence for rhythms in the ranges reported by Kyriacou and Hall (the grey, red, and blue bands in Figure 3). Recordings of males carrying *period* mutations and of *D. simulans* males displayed a similar distribution of significant Lomb-Scargle peaks (Figure 4a). These high frequency rhythms are distributed across a wide frequency range and probably result from modulation of inter-pulse intervals by males over short time scales (c.f. Figure 1b,c). Recordings of *D. simulans* males displayed a weak tendency to produce relatively lower frequency periodicity than did *D. melanogaster* recordings (Figure 4a), which is opposite to the observations of Kyriacou and Hall (Figure 1e) [6,9,10]. There is no evidence that these rhythms are constrained to a narrow frequency range. Thus, statistical analysis of long recordings of courtship song provides no support for the existence of KH cycles.

KH cycles observed by previous authors might have resulted from the specific analysis techniques or sampling procedures employed in earlier studies. In different papers, authors used different spectral analysis techniques to search for rhythms, so it is unlikely that KH cycles resulted from any specific analytical technique. However, there are two parameters that are common to most published studies: inter-pulse interval data were binned into 10 s intervals and most recordings were approximately 5 min long or shorter (Additional file 1: Table S1). In addition, most studies did not incorporate a robust method of assessing the statistical significance of rhythmicity [8]. Thus, often the periodogram peak with greatest power was taken as the best estimate of rhythmicity. Sampling just the peaks representing the greatest power in the Lomb-Scargle periodograms did not obviously alter the distribution of frequencies from the sample of all peaks with P < 0.01 (Figure 3c). However, 10 s binning of inter-pulse intervals dramatically altered the distribution of strongest signals in the Lomb-Scargle periodograms, forcing many values to be within the frequency range reported for KH cycles (Figure 3d, compare with colored columns), although few of these signals were statistically significant. This truncation of the high frequency range of the spectrogram resulted simply from the fact that the highest frequency that can be estimated in any time series is equal to half the sampling rate, or 0.05 Hz (1/20 s) for 10 s bins [35].

To more precisely replicate the data used in previous studies, I analyzed the first five minutes of song following the first train of pulse song. I also sampled non-overlapping 5 min clips from across the entire duration of recordings. Both samples of 5 min of song resulted in poor estimation of low frequency rhythms, forcing approximately 65% of the maximal rhythms to be within the range reported for KH cycles (Figure 3d,e). This is similar to the proportion of songs with KH cycles that was reported in the later publications from Kyriacou and Hall (Figure 2a), suggesting that these reported KH rhythms could be fully explained as artifacts of binning short songs. In addition to these manipulations, I also tested many other manipulations of the data described in [30], including multiple methods for estimating values for empty bins. None of these other manipulations had a large effect on the distributions illustrated in Figure 4 (not shown).

The pattern observed for the wild-type Canton-S strain of *D. melanogaster* was also observed for a wild-type strain of *D. simulans* and for the three *period* mutations (Figure 4a). Binning short songs constrained the maximal rhythmicity close to the range reported by Kyriacou and Hall (Figure 4a). It is important to note that binning the data, but not shortening the songs, reduced the power of this rhythmicity, resulting in much lower statistical significance for the maximal periodogram peaks (Figure 4a). In fact, fewer than two percent of the maximal periodogram peaks observed in binned data reached a threshold for statistical significance of *P* <0.05. That is, binning did not generate statistically significant rhythms, it simply constrained the maximal peak of periodograms so that they were more likely to fall in the range reported by Kyriacou and Hall. Since most previous studies did not incorporate robust methods of estimating the statistical significance of the maximal peaks, it is likely that their reported maximal peaks were, like the ones I found, not statistically significant.Randomizing inter-pulse interval data produced patterns of maximal periodogram peaks similar to the original data, but the statistical significance of the peaks was much lower on average in the randomized data than in the original data (Figure 4b). This provides further evidence that the statistically significant high frequency rhythms detected in the original data reflect real fluctuations in inter-pulse intervals on the order of seconds.

## Discussion

While it may be impossible to prove that a phenomenon does not exist, the foundations of science rest upon repeatable observations. KH cycles cannot be replicated. Perhaps more importantly, we now have an explanation for why Kyriacou, Hall and colleagues observed courtship song rhythms in a particular frequency range. Periodogram peaks with periods on the order of 60 s are an artifact of binning data from short songs into 10 s intervals. Most of this apparent rhythmicity does not reach any sensible level of statistical significance. KH cycles appear to represent fluctuations of noise, which is supported by their similarity to patterns generated by randomized data.

My observations do not explain why Kyriacou and Hall observed that different genotypes appeared to produce KH cycles with slightly different average periods nor why females of different species appeared to prefer KH cycles with periods matching that of their own species [9]. One possibility is that the observed average differences between genotypes represented random samples from the full distribution of rhythms generated artificially by the analysis techniques, and that by using relatively small sample sizes (N = 8 ± 3.9 (mean ± standard deviation); Additional file 2: Table S2), Kyriacou and Hall fortuitously observed frequencies consistent with their hypotheses. This possibility is emphasized by the extensive overlap seen in the reconstructed ranges of periods reported in earlier studies (Figure 2b). This small-sample-size effect may have been exacerbated by the exclusion of many songs that did not exhibit rhythmicity (Figure 2a, Additional file 2: Table S2), which was common practice in all papers on this subject.

## Conclusions

For decades, the work of Kyriacou and Hall has appeared far ahead of its time and their evidence for the role of the *period* gene in behavioral evolution has stood as a shining example of how genetic evolution can generate a species-specific behavior. Unfortunately, it appears highly likely that this example is not true. This case highlights some of the challenges inherent in identifying the genetic causes of behavioral evolution. Behavior is often variable on multiple time scales and as a result of multiple genetic and environmental influences. Scoring behavior and detecting species-specific differences requires objective methods for quantifying behavior. Given that most behaviors are likely to have evolved as the result of evolution at multiple loci [36], large samples of individuals must be assayed. Satisfactory methodologies have only recently become available for scoring a handful of fly behaviors [3,37-40]. There is now at least some hope that these questions can be addressed in a rigorous manner.

## Methods

Virgin males and females were collected from the Canton-S strain of *D. melanogaster* and from a wild-type strain of *D. simulans* collected in Princeton, NJ, USA in 2009. Individual males were isolated in single wells of a 96-well Scienceware™ 96 deep-well plate (Bel-Art, Wayne, NJ, USA) containing approximately 1 mL of standard cornmeal-based fly food covered with an AirPore Tape Sheet (Qiagen, Hilden, Germany). Males were aged for 5 to 10 days in a 25°C incubator with a 12 hour:12 hour light:dark cycle. Females were group housed in standard *Drosophila* vials and maintained in the same incubator with males for 1 to 3 days prior to pairing with males for recording.

We built 20 × 10 × 3 mm recording chambers from acrylic, which matched the specifications reported in [6] and which could be fitted in our 32-channel recording system [3]. Data were collected and segmented as reported previously [3].

### Data analysis

Following Kyriacou and Hall, inter-pulse intervals were defined as times between pulse events that were between 15 and 65 ms long. I excluded songs with fewer than 1,000 pulses and time series were initiated at the pulse train that contained at least 10 inter-pulse intervals, as described in [30]. Data were analyzed using the Lomb-Scargle periodogram with the MATLAB script ‘lomb’ written by Saragiotis and available from [41]. Data were binned by calculating the mean inter-pulse interval for 10 s intervals that each contained at least 10 pulses, as described in [30]. Five-minute clips were generated by dividing 45 min recordings into 5 min segments. For the randomization test, all times between pulses were calculated (including the pauses between pulse trains), and these values were shuffled without replacement. This procedure generated time series that resembled real song, but both the initiation time of pulse trains and the inter-pulse intervals were shuffled. All other analyses were performed using custom MATLAB scripts. The raw song data are available at [42]. The segmented inter-pulse intervals for all individuals are available as individual CSV files at [43]. Complete details of the analysis pipeline, including step-by-step instructions to replicate the results reported here, are provided in a file called AnalysisPipeline.rtf together with all required MATLAB scripts in the zipped folder available at [44].

## Competing interests

The author declares that he has no competing interests.

## Supplementary Material

Additional file 1: Table S1 Statistics of data underlying reported KH cycles from previous publications. Click here for file

Additional file 2: Table S2 Values of reported KH cycles from previous publications, with back-calculated standard deviations. Click here for file

Additional file 3: Figure S1 Power of the Lomb-Scargle periodogram to detect simulated KH cycles in courtship song time series. Click here for file
