# Supplementary material for: Reported Drosophila courtship song rhythms are artifacts of data analysis
Source: BMC Biol. 2014 Jun 26;12:38. doi: 10.1186/1741-7007-12-38 (PMC4071150; doi:10.1186/1741-7007-12-38)
Supplement: Additional file 1: Table S1 — Statistics of data underlying reported KH cycles from previous publications. [file 1741-7007-12-38-S1.docx]

Supplementary Table 1. Statistics of all reports of searches for KH cycles in courtship song.

| Number significant at 5% level^[[1]](#endnote-1)^ | Sample size | Song length | Genotype | Data pooled? | Detection method | Date | Publication |
| --- | --- | --- | --- | --- | --- | --- | --- |
| 42 | 44 | ≤ 5-6 min | *D. melanogaster* | 10s bins | Nonlinear regression | 1980 | [1] |
| 22 | 24 | ≤ 5-6 min | *per* altered rhythm alleles | 10s bins | Nonlinear regression | 1980 | [1] |
| 89 | 98 | ≤ 5-6 min | *per* alleles in sexually transformed flies | 10s bins | Nonlinear regression | 1980 | [1] |
| 4 | 5 | ≤ 5-6 min | *D. simulans* | 10s bins | Nonlinear regression | 1980 | [1] |
| 5 | 7 | 4-7 min | *per* rescue fragment 14.6 | 10s bins | Nonlinear regression | 1984 | [2] |
| 0 | 2 | 4-7 min | *per* rescue fragment 9.8 | 10s bins | Nonlinear regression | 1984 | [2] |
| 4 | 5 | 4-7 min | *per* rescue fragment 8.0 | 10s bins | Nonlinear regression | 1984 | [2] |
| 3 | 4 | 4-7 min | *per* + controls | 10s bins | Nonlinear regression | 1984 | [2] |
| 18 | 20 | ≤5-6 min | *D. melanogaster* | 10s bins | Nonlinear regression | 1986 | [3] |
| 12 | 19 | ≤5-6 min | *D. simulans* | 10s bins | Nonlinear regression | 1986 | [3] |
| 23 | 34 | ≤5-6 min | *D. melanogaster* X *D. simulans* hybrids | 10s bins | Nonlinear regression | 1986 | [3] |
| 51 | 74 | ≤5-7 min | *per* gene transformants | 10s bins | CLEAN and others | 1991 | [4] |
| 24 | 37 | ≤5-7 min | *D. melanogaster* | 10s bins | CLEAN and others | 1991 | [4] |
| 11 | 15 | ≤5-7 min | *D. simulans* | 10s bins | CLEAN and others | 1991 | [4] |
| 7-25^[[2]](#endnote-2)^ | 40 | 10 min | *D. melanogaster* and *per* mutants | 1, 5, 10s bins | Modified fourier transform | 1998 | [5] |
| 1^[[3]](#endnote-3)^ | 61 | 14 min | *D. melanogaster* strains | No | Lomb-Scargle Periodogram | 2013 | [6] |
| 2 | 154 | 43 min | *D. melanogaster* strains, *per* alleles, *D. simulans* | No | Lomb-Scargle Periodogram | 2014 | This study |

1. Different methods were used to assess significance. In the early studies of Kyriacou and Hall, the significance of rhythms was assessed with an F test, which is probably inappropriate, given the fact that residuals from the non-linear regressions are probably not distributed normally [7,8]. [↑](#endnote-ref-1)
2. Data were binned in three different ways and analyzed by two different methods. [↑](#endnote-ref-2)
3. In original paper, 29 songs were reported that contained at least one periodogram peak that met significance in the frequency range of 0.016 – 0.022 HZ. To be consistent with previous reports, here I report number of songs with maximum power that met significance in the frequency range of 0.016 – 0.022 HZ. [↑](#endnote-ref-3)
