# Supplementary material for: Reported Drosophila courtship song rhythms are artifacts of data analysis
Source: BMC Biol. 2014 Jun 26;12:38. doi: 10.1186/1741-7007-12-38 (PMC4071150; doi:10.1186/1741-7007-12-38)
Supplement: Additional file 2: Table S2 — Values of reported KH cycles from previous publications, with back-calculated standard deviations. [file 1741-7007-12-38-S2.docx]

Supplementary Table 2. Values of reported KH Cycles means and standard errors, along with back calculated standard deviations.

| **Paper** | **Date** | **Table** | **Genotype** | **Period Mean** | **Period SE** | **N** | **Period SD** | **Notes** |  |
| --- | --- | --- | --- | --- | --- | --- | --- | --- | --- |
| K&H 1980 | 1980 | 1 | Canton-S | 54 | 0.8 | 9 | 2.4 |  |  |
| K&H 1980 | 1980 | 1 | yellow | 56.7 | 2.1 | 8 | 5.9 |  |  |
| K&H 1980 | 1980 | 1 | Oregon-K | 54.1 | 1.8 | 9 | 5.4 |  |  |
| K&H 1980 | 1980 | 1 | y sn[3] m | 54 | 2.4 | 6 | 5.9 |  |  |
| K&H 1980 | 1980 | 1 | XX/Y;tra/tra | 53.4 | 1.9 | 4 | 3.8 |  |  |
| K&H 1980 | 1980 | 1 | X/Y;tra/tra | 53.2 | 1 | 4 | 2.0 |  |  |
| K&H 1980 | 1980 | 1 | X/Y;tra/TM6 | 53.1 | 1.1 | 4 | 2.2 |  |  |
| K&H 1980 | 1980 | 2 | per[s] | 41.5 | 1 | 12 | 3.5 | 24 per[s] and per[l] measured. N divided by 2 for each. | |
| K&H 1980 | 1980 | 2 | per[l] | 82.1 | 3.3 | 12 | 11.4 |  |  |
| K&H 1980 | 1980 | 2 | per[o] | NaN | NaN | ? |  |  |  |
| K&H 1980 | 1980 | 3 | per[s]/per+ | 43.1 | 1.4 | 12 | 4.8 |  |  |
| K&H 1980 | 1980 | 3 | per[l]/per+ | 53.2 | 1.1 | 14 | 4.1 |  |  |
| K&H 1980 | 1980 | 3 | per[s]/per[l] | 52.2 | 1.2 | 13 | 4.3 |  |  |
| K&H 1980 | 1980 | 3 | per[o]/per[l] | 39.7 | 0.7 | 14 | 2.6 |  |  |
| K&H 1980 | 1980 | 3 | per[o]/per[s] | 32.4 | 1.2 | 12 | 4.2 |  |  |
| K&H 1980 | 1980 | 3 | per[o]/per[l] | 54.7 | 1.2 | 12 | 4.2 |  |  |
| K&H 1980 | 1980 | 3 | per+/Df | 39.4 | 0.7 | 7 | 1.9 |  |  |
| K&H 1980 | 1980 | 3 | per[s]/Df | 33.7 | 1.2 | 8 | 3.4 |  |  |
| K&H 1980 | 1980 | 3 | per[l]/Df | 51.7 | 1.9 | 6 | 4.7 |  |  |
| Zehring et al 1984 | 1984 | 1 | Transformant 14.6 | 68.2 | 7.3 | 7 | 19.3 |  |  |
| Zehring et al 1984 | 1984 |  | Transformant 8.0 | 79.4 | 9 | 4 | 18.0 |  |  |
| Zehring et al 1984 | 1984 |  | per+ | 58.8 | 1.5 | 3 | 2.6 |  |  |
| KH 1989 | 1989 | 1 | yellow | 58.5 | 3 | 9 | 9.0 |  |  |
| KH 1989 | 1989 | 1 | CantonS | 54 | 0.8 | 9 | 2.4 |  |  |
| KH 1989 | 1989 | 1 | D. simulans | 33.2 | 1 | 5 | 2.2 |  |  |
| KH 1989 | 1989 | 1 | D. simulans LHR | 38.7 | 0.8 | 2 | 1.1 |  |  |
| KH 1989 | 1989 | 1 | X[sim]/Y[mel | 39 | 0.8 | 8 | 2.3 |  |  |
| KH 1989 | 1989 | 1 | X[mel-per+]/Y[sim] | 56.5 | 1.9 | 6 | 4.7 |  |  |
| KH 1989 | 1989 | 1 | X[mel-per[l]]/Y[sim] | 75.4 | 3.9 | 5 | 8.7 |  |  |
| KH 1989 | 1989 | 1 | X[mel-per[o]]/Y[sim] | 141.6 |  | 1 | 0.0 |  |  |
| Wheeler 1991 | 1991 | 1 | 13.2m-TGm | 58.6 | 2.6 | 10 | 8.2 | Periods for individuals assessed as displaying rhythmicity. Transformant fragment 13.2 and threonine-glycine repeat come from melanogaster (m) or simulans (s) | |
| Wheeler 1991 | 1991 | 1 | 13.2s-TGs | 37 | 3 | 12 | 10.4 | Periods for individuals assessed as displaying rhythmicity. Transformant fragment 13.2 and threonine-glycine repeat come from melanogaster (m) or simulans (s) | |
| Wheeler 1991 | 1991 | 1 | 13.2m-TGs | 34.7 | 2.3 | 17 | 9.5 | Periods for individuals assessed as displaying rhythmicity. Transformant fragment 13.2 and threonine-glycine repeat come from melanogaster (m) or simulans (s) | |
| Wheeler 1991 | 1991 | 1 | 13.2s-TGm | 58.2 | 5 | 12 | 17.3 | Periods for individuals assessed as displaying rhythmicity. Transformant fragment 13.2 and threonine-glycine repeat come from melanogaster (m) or simulans (s) | |
| Wheeler 1991 | 1991 | 1 | D. melanogaster (Chieti-V) | 52.7 | 3.7 | 9 | 11.1 |  |  |
| Wheeler 1991 | 1991 | 1 | D. melanogaster (Oxford) | 54.5 | 2.6 | 6 | 6.4 |  |  |
| Wheeler 1991 | 1991 | 1 | D. melanogaster (Canton-S) | 53.6 | 5.9 | 9 | 17.7 |  |  |
| Wheeler 1991 | 1991 | 1 | D. simulans (Australia) | 39.4 | 2.9 | 5 | 6.5 |  |  |
| Wheeler 1991 | 1991 | 1 | D. simulans (Kenscoff) | 33.8 | 2.6 | 3 | 4.5 |  |  |
| Wheeler 1991 | 1991 | 1 | D. simulans (Georgetown) | 39.1 | 4.7 | 3 | 8.1 |  |  |
|  |  |  |  |  |  |  |  |  |  |
|  |  |  |  |  |  |  |  |  |  |
|  |  |  |  |  |  |  |  |  |  |
|  |  |  |  |  |  |  |  |  |  |
