# Supplementary material for: Reported Drosophila courtship song rhythms are artifacts of data analysis
Source: BMC Biol. 2014 Jun 26;12:38. doi: 10.1186/1741-7007-12-38 (PMC4071150; doi:10.1186/1741-7007-12-38)
Supplement: Additional file 3: Figure S1 — Power of the Lomb-Scargle periodogram to detect simulated KH cycles in courtship song time series. [file 1741-7007-12-38-S3.pdf]

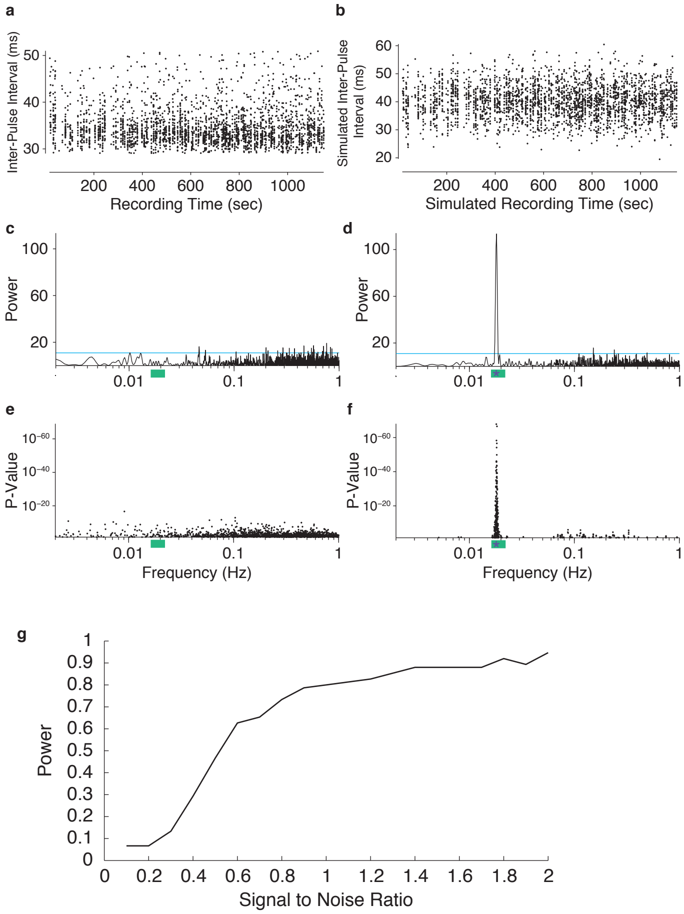

Supplementary Figure 1. Statistical analysis of inter-pulse interval rhythms. (a) An example of inter-pulse intervals produced by a single individual during a single recording session. (b) An example of simulated inter-pulse intervals with a KH cycle of 0.018 Hz, a SNR = 1, and sampling times derived from the data in panel a. (c,d) The Lomb-Scargle periodograms for the real data shown in panel a (c) and for the simulated data shown in panel b (d). Horizontal cyan lines indicate power where P = 0.05. (e, f) P-values of the local peaks in the Lomb-Scargle periodograms of inter-pulse interval for 75 recordings of *D. melanogaste*r (e) and for 75 simulated datasets with a SNR = 1 (f) over the range of 0 to 1 Hz. Only P-values below 0.05 are plotted. The green bars below the axes in c-f mark the range of 0.016 to 0.22 Hz. The asterisks within the green bars in d and f indicate 0.018 Hz, the frequency used in these simulations. The naturally skewed distribution of the real data, for example in panel a, differs from the distribution in the simulated data. We found, however, that culling the data to generate Gaussian distributed inter-pulse interval data resulted in even fewer significant peaks in the periodograms than shown in panel e and no obvious clustering of peaks in any particular frequency range (not shown). (g) Power to detect significant peaks (P < 0.05) in the range of 0.016 – 0.022 Hz in the Lomb-Scargle periodograms from simulated data as the SNR varies from 0.1 to 2. Panels a-bfare reproduced from Fig. 5 of ref [7] and panel g is reproduced from Supplementary Figure S6 of ref [7]
